# Importance of Social and Cultural Factors for Attitudes, Disclosure and Time off Work for Depression: Findings from a Seven Country European Study on Depression in the Workplace

**DOI:** 10.1371/journal.pone.0091053

**Published:** 2014-03-12

**Authors:** Sara Evans-Lacko, Martin Knapp

**Affiliations:** 1 Health Service and Population Research Department, King's College London, Institute of Psychiatry, London, United Kingdom; 2 London School of Economics and Political Science, London, United Kingdom; Shanghai Mental Health Center, Shanghai Jiao Tong University School of Medicine, China

## Abstract

**Objectives:**

Depression is experienced by a large proportion of the workforce and associated with high costs to employers and employees. There is little research on how the social costs of depression vary by social and cultural context. This study investigates individual, workplace and societal factors associated with greater perceived discomfort regarding depression in the workplace, greater likelihood of employees taking time off of work as a result of depression and greater likelihood of disclosure of depression to one's employer.

**Methods:**

Employees and managers (n = 7,065) were recruited from seven European countries to participate in the IDEA survey. Multivariable logistic regression models were used to examine associations between individual characteristics and country contextual characteristics in relation to workplace perceptions, likelihood of taking time off work and disclosing depression to an employer.

**Results:**

Our findings suggest that structural factors such as benefit systems and flexible working hours are important for understanding workplace perceptions and consequences for employees with depression. However, manager responses that focus on offering help to the employee with depression appear to have stronger associations with positive perceptions in the workplace, and also with openness and disclosure by employees with depression.

**Conclusion:**

This study highlights the importance of individual, workplace and societal factors that may be associated with how people with depression are perceived and treated in the workplace, and, hence, factors that may be associated with openness and disclosure among employees with depression. Some responses, such as flexible working hours, may be helpful but are not necessarily sufficient, and our findings also emphasise the importance of support and openness of managers in addition to flexible working hours.

## Introduction

According to the most recent Global Burden of Disease 2010 statistics, depression ranks as a leading cause of disability [1], [2] and according to the World Health Organization, is the leading cause of disability worldwide, influencing 350 million people [3]. Its early onset and chronic nature have significant consequences for forgone education and employment prospects, making it second in terms of years lost to disability. In Europe, it is estimated that depression accounts for 7.2% of the overall disease burden [4], with associated costs totalling around €92 billion and affecting 30 million EU citizens [4]–[6].

By far, the greatest contributor to the overall economic impact of depression is loss in productivity [7], [8]. For example, population survey data from the USA estimate annual human capital loss to be $4,426 per employed person with major depression [9]. A more recent study of individuals using secondary mental health services in Sweden estimated the mean annual per person productivity losses to be €15,206 [7]. The impact of depression on productivity is related to illness severity, with comorbidity, chronicity and severity all contributing towards worse outcomes [10]; but, even mild depression is associated with significant productivity losses [11]. At the population level, major depression has a greater impact on workplace absenteeism than other chronic mental and physical disorders [12]. In addition to absenteeism, presenteeism is especially significant for people with depression and may be associated with costs five times greater than those due to absenteeism [13], [14].

The strong evidence for links between depression and impaired work performance contrasts with beliefs reported by employers. A recent survey of 500 employers in the UK showed that nearly half of respondents felt that employees “suffering from stress are able to work effectively at all time points” [15]. Additionally, although most employers reported awareness and understanding of workplace policies, almost half (42%) thought “they were primarily designed to help their organisation avoid litigation.” A positive work environment and access to appropriate and effective treatment may mitigate the risk and impact of depression in the workplace [16], [17]; however, many employees report experiences of, or fear of stigma and discrimination at work which may exacerbate their distress and impede help-seeking. A global survey of individuals with major depressive disorder found that 71% of respondents preferred to conceal their depression from others in the workplace and 47% anticipated discrimination in finding or keeping a job due to their diagnosis [18].

The workplace context and attitudes of employees and managers may be important for how individuals experience depression in the workplace or make decisions around disclosure or taking time off from work. Societal beliefs, cultural context, national and local policies, and employment and related regulations may also influence decisions made by employers or reactions from employees in response to an employee with depression. In this study we investigate individual, workplace and societal factors that might be associated with greater perceived discomfort regarding depression in the workplace. We examine whether and how these factors are associated with: (i) greater likelihood of employees taking time off work as a result of depression; and (ii) greater likelihood of disclosure of depression to their employer.

## Methods

### Data source

For this study, we performed secondary data analysis on the IDEA (Impact of Depression in the Workplace in Europe Audit) survey data which were collected to gain insight on levels of awareness of the identification, impact and burden of the cognitive symptoms of depression across Europe for European Depression Day. Participants were recruited for the IDEA survey through an online market research panel. Before joining the panel, participants went through a screening process to validate their personal data which included: removal of duplicates, validation of name and surname through name/gender match or mismatch/misspelling as compared to library of names, country validation based on IP address (internet protocol address used to identify unique users), validation of town and zip/postal code according to official lists, checking for valid correlations between sociodemographic data (gender, age of parents and children) and validation of contact information. Individuals who worked in advertising and/or market research, and those aged under 16 years old were excluded.

Selected panel members were invited to participate in the survey through Ipsos MORI (www.ipsos-mori.com/) if they were employed and they resided in one of seven participating countries. Response rates varied by country and were (from highest to lowest): France (38.5%), Italy (38.1%), Spain (23.6%), Germany (22.4%), UK (16.4%), Turkey (13.7%) and Denmark (8.2%). Questionnaires were collected from approximately 1,000 respondents per country.

### Measures

*Sociodemographic information* included age band (16–24, 25–44, and 45–64 years), gender, highest education level (secondary school or earlier, professional qualification, higher education (below university, university degree)), marital status (single, married/cohabitating, divorced/separated, widowed) and working status (full-time, part-time, previously employed in the last 12 months).

*Previous diagnosis of depression* was determined via self-report by asking respondents: Have you ever personally been diagnosed as having depression by a doctor/medical professional? For respondents who responded positively to the question about a diagnosis of depression, two follow-up items were then asked: (1) “Have you personally ever taken time off work because of your depression?” and (2) “Still thinking about the last time you were off work due to depression, did you tell your employer that the reason you needed to take time off work was because of your depression?”

### Country variables

We used data from the IDEA survey to describe the overall population prevalence of managerial responses to employees with depression. Managers who said that they had one or more employees with depression in the past were asked how they responded to the employee. Potential responses included: (i) Offered a different work pattern (flexible working, leave etc.); (ii) avoided talking to them about it; (iii) encouraged them to talk to a healthcare professional and (iv) discussed with them and asked if there was anything I [the manager] could do to help.

Estimates of the country replacement ratio were obtained via the OECD [19]. In this case, replacement ratio refers to gross replacement rates by level of individual earnings specifically including employment insurance and unemployment assistance benefits. Higher replacement rates are associated with a more generous benefits scheme.

### Statistical analysis

Sociodemographic characteristics (gender, age, marital status, education and working status) and attitudes and beliefs about depression were analysed for respondents with versus without a prior diagnosis of depression. A small proportion of respondents (1.7%) refused to answer the question regarding depression diagnosis. Individuals who refused vs. did not refuse to answer were compared based on sociodemographic characteristics and there were no significant differences except that individuals with a university education were more likely to refuse answering the question (p = 0.046). Reported prevalence of depression diagnoses and overall attitudinal and welfare/benefit characteristics are then presented by country.

Among individuals who reported a prior diagnosis of depression, two multivariable logistic regression models were used to examine (i) factors associated with a greater likelihood of employees taking time off work as a result of depression and (ii) likelihood of disclosure of depression to one's employer. A third multivariable logistic regression model investigated factors associated with greater perceived discomfort regarding depression in the workplace, now looking at all respondents. Country contextual characteristics were computed as an average rating for each country across respondents, and each variable was standardized (i.e., z score was computed). Post-stratification weights, based on gender, age and region, which were aligned with nationally representative figures, were used in all analyses. We used generalized estimating equations (GEE) with robust variance estimates to model within-country correlations [20]. We selected GEE instead of mixed regression models as we were interested in understanding the influence of overall cultural factors rather than individual country level effects. Thus, a population average model was more appropriate for our research question. As GEE is a non-likelihood based method, Pan's QIC (quasi-likelihood under the independence model criterion) was used for variable selection and selecting the working correlation matrix. QIC is a statistic which generalizes AIC (Akaike Information Criterion) to GEE models by replacing likelihood estimation with quasi-likelihood estimation and making adjustments for the penalty term. A lower QIC value indicates better model fit. [21]. All analyses were carried out using SAS version 9.3.

### Ethics statement

This study was classified as exempt by the King's College London, Psychiatry, Nursing, and Midwifery Research Ethics Subcommittee as this was secondary data and was fully anonymised. Data collection was performed independently by Ipsos MORI in accordance with the standards of ESOMAR, AIMRI and EFAMRO in Europe and are in line with the data protection act 1998. Data were collected as part of a market research survey and are hosted with the market research agency Ipsos MORI. All data for the market research survey are anonymous and did not include any personal information. No minors or children were involved in the study and written consent was obtained. Data can be provided upon request.

## Results

### Participant characteristics

Socio-demographic and attitudinal characteristics of employees who did vs. did not report a previous diagnosis of depression are described in table 1. Employees who reported a diagnosis of depression were more likely to be female, divorced and working part-time. Individuals who reported never having a diagnosis of depression were more likely to be married, in the youngest age group (16–24) and working full-time. In terms of attitudes, individuals with a diagnosis of depression were more likely to rank depression as the most disabling illness (relative to cardiovascular problems, serious deafness/loss hearing, depression, alcoholism/alcohol abuse, and cerebrovascular disease). Moreover, although individuals with and without depression had similar rankings in terms of symptoms associated with depression, individuals with depression were more likely to endorse the prevalence of all symptoms associated with depression as higher than individuals without a diagnosis of depression. Both groups were more likely to agree that affective symptoms were associated with depression compared to cognitive symptoms.

**Table 1:** Descriptive characteristics of people with and without depression in the workplace.

Weighted percentages (95% Confidence interval)			
	Individuals reporting experience of depression n = 1,412	Individuals reporting no experience of depression n = 5,534	p-value
**Socio-demographic characteristics**			
Gender			<0.001
Male	42.4 (39.8, 44.9)	57.8 (56.4, 59.1)	
Female	57.7 (55.1, 60.2)	42.2 (40.9, 43.5)	
Age (years)			<0.001
16–24	7.1 (5.3, 8.9)	10.8 (9.8, 11.8)	
25–44	51.3 (48.5, 54.0)	51.4 (50.0, 52.8)	
45–64	41.7 (38.9, 44.4)	37.8 (36.4, 39.2)	
Marital status			
Single	26.3 (23.8, 28.7)	26.4 (25.3, 27.6)	
Married/cohabitating	59.7 (56.7, 61.8)	64.9 (63.7, 66.2)	
Divorced Separated	12.7 (10.9, 14.4)	7.1 (6.4, 7.8)	
Widowed	1.3 (0.7,1.7)	0.6 (0.4, 0.8)	
Refused	0.6 (0.2, 1.1)	0.9 (0.7, 1.2)	
Education			<0.001
Secondary school or earlier	7.7 (6.3, 9.1)	7.4 (6.7, 8.1)	
Professional qualification	21.3 (19.2, 23.5)	22.3 (21.2, 23.4)	
Higher education (below university)	20.5 (18.4, 22.6)	19.0 (18.0, 20.1)	
University degree	36.9 (33.4, 39.4)	36.8 (35.6, 38.1)	
Refused	13.5 (11.7, 15.3)	14.4 (13.5, 15.3)	
Working status			<0.001
Full time	71.0 (68.6, 73.3)	77.2 (76.1, 78.4)	
Part time	23.4 (21.2, 25.6)	17.6 (16.6, 18.6)	
Previously employed in last 12 months	5.7 (4.5, 6.9)	5.2 (4.6, 5.8)	
**Mental health related attitudes**			
Responses to attitude items			<0.001
Ranked depression as most disabling	21.2 (18.9, 23.4)	14.6 (13.7, 15.5)	
Ranked depression as least disabling	13.6 (11.8, 15.5)	16.7 (15.7, 17.6)	
Beliefs about symptoms of depression			<0.001
Low mood	92.7 (91.4, 94.1)	87.6 (86.7, 88.6)	
Loss of interest in daily activities	83.4 (81.3, 85.4)	55.8 (54.5, 57.2)	
Trouble sleeping/insomnia	81.9 (79.7, 84.0)	71.7 (70.4, 72.9)	
Crying for no reason	74.7 (72.3, 77.1)	71.7 (70.4, 73.0)	
Trouble concentrating	67.3 (64.7, 69.9)	53.8 (52.3, 55.2)	
Changes in weight/appetite	65.2 (62.5, 67.9)	53.1 (51.7, 54.5)	
Difficulty planning day to day activities	66.0 (63.4, 68.6)	55.8 (54.4, 57.2)	
Indecisiveness	55.4 (52.7, 58.1)	40.8 (39.4, 42.2)	
Forgetfulness	47.0 (44.2, 49.7)	28.9 (27.6, 30.2)	

p-values show significance level of Pearson's Chi square test.

### Country averages

Table 2 describes weighted country averages for employee-reported depression and manager responses to an employee with depression across the seven participating countries. There are some differences by country. Female and male respondents from Italy were less likely to report having a diagnosis of depression compared to respondents from Great Britain and from Turkey. Managers in Denmark were less likely than managers in France, Germany, Italy, Spain and Turkey to say that they would avoid employees with depression, and more likely than managers in Germany and Italy to say that they would offer help to employees. Managers in France and Spain were the most likely to recommend that the employee seek help from a healthcare professional.

**Table 2:** Prevalence of depression and reaction by managers to employees with depression by country.

Weighted percent (95% Confidence Interval)								
	Denmark (n = 1013)	France (n = 1003)	Germany (n = 1001)	Great Britain (n = 1002)	Italy (n = 1017)	Spain (n = 1008)	Turkey (n = 1021)	p-value
**Employee clinical characteristics**								
Prevalence of employees reporting a diagnosis of depression								<0.001
Females	20.7 (16.7, 24.7)	23.2 (19.4, 27.0)	24.7 (20.8, 28.7)	36.1 (31.6, 40.5)	17.2 (13.5, 20.9)	26.1 (22.1, 30.2)	32.4 (27.3, 37.5)	
Males	18.1 (14.1, 22.1)	15.9 (12.7, 19.1)	14.5 (11.5, 17.6)	18.8 (15.5, 22.0)	9.2 (6.9, 11.6)	17.5 (14.4, 20.7)	19.4 (15.5, 23.2)	
Days off of work due to depression								<0.001
0	51.9 (42.5, 61.3)	50.9 (43.5, 58.4)	44.4 (36.67, 52.1)	46.8 (40.4, 53.2)	56.6 (47.4, 65.9)	53.2 (46.1, 60.3)	76.6 (69.7, 83.5)	
1–10	14.2 (7.8, 20.7)	13.1 (8.1, 18.2)	9.8 (5.2, 14.5)	13.5 (9.1, 17.9)	24.0 (16.0, 32.0)	17.3 (11.9, 22.8)	7.1 (3.8, 10.3)	
11–20	7.9 (3.9, 12.0)	12.0 (7.1, 16.8)	13.7 (8.3, 19.1)	7.6 (4.2, 11.0)	7.8 (2.8, 12.8)	6.1 (2.7, 9.5)	4.2 (1.4, 7.0)	
20+	25.9 (18.5, 33.4)	24.0 (17.6 (30.4)	32.1 (24.8, 39.3)	32.1 (26.1, 38.1)	11.5 (5.5, 17.5)	23.4 (17.4, 29.4)	12.1 (6.0, 18.3)	
**Manager-reported reactions to an employee with depression**								
Offered help	51.2 (46.7, 55.8)	44.5 (40.3, 48.7)	38.7 (33.6, 43.9)	53.0 (48.3, 57.8)	39.7 (35.0, 44.4)	56.2 (51.8, 60.6)	55.2 (49.3, 61.1)	<0.001
Offered flexible working pattern	12.5 (9.7, 15.4)	10.0 (7.5, 12.5)	8.5 (5.6, 11.4)	11.9 (8.8, 14.9)	7.5 (4.9, 10.0)	9.3 (6.7, 11.8)	13.6 (10.0, 17.1)	<0.001
Encouraged talking to a healthcare professional	29.6 (25.3, 33.9)	41.5 (37.3, 45.6)	28.1 (23.4, 32.9)	39.8 (35.1, 44.4)	38.5 (33.8, 43.1)	44.9 (40.5, 49.3)	35.5 (30.1, 40.9)	<0.001
Avoided talking about it	1.8 (0.7, 2.9)	5.3 (3.4, 7.2)	7.8 (4.9, 10.6)	3.3 (1.6, 4.9)	12.4 (9.3, 15.6)	6.1 (4.0, 8.2)	9.3 (5.7, 12.8)	<0.001
**Characteristics of benefits system by country**								
Replacement ratio (OECD)^a^	40	49	41	29	23	42	22	<0.001

a In this case, replacement ratio refers to gross replacement rates by level of individual earnings specifically including employment insurance and unemployment assistance benefits obtained via OECD.

p-values show significance level of Pearson's Chi square test.

Table 3 describes the individual and country contextual characteristics associated with greater perceived discomfort in relation to employees with depression in the workplace. In terms of individual characteristics, females were less likely than males to perceive discomfort in the workplace. In terms of country contextual characteristics, on average living in a country with a greater prevalence of managers saying that they offered help to an employee with depression was associated with less perceived discomfort. On average, living in a country with a greater prevalence of managers avoiding talking to the employee about depression and a greater prevalence of managers saying that they offered a flexible working pattern was associated with greater perceived discomfort. On average, living in a country with a more generous benefits scheme (i.e., higher replacement ratio) was associated with greater perceived discomfort regarding depression in the workplace.

**Table 3:** Individual, manager and country contextual characteristics associated with greater likelihood of endorsing that someone in the workplace with depression would make other employees feel uncomfortable a (Multivariable logistic regression, n = 7,065).

	Adjusted GEE parameter estimates Odds Ratio (95% CI)
**Individual characteristics**	
Gender	
Female	**0.70 (0.61, 0.79)** **
Male	Reference
Age	
45–64	0.97 (0.65, 1.46)
25–44	0.87 (0.62, 1.21)
16–24	Reference
University education	
Yes	1.04 (0.85, 1.26)
No	Reference
Diagnosed with depression	
Yes	1.02 (0.95, 1.08)
No	Reference
Working in a larger company	1.03 (0.98, 1.08)
**Country contextual characteristics**	
Country prevalence of manager reactions to someone with depression	
Offered help to employee	**0.73 (0.65, 0.82)** ***
Offered flexible working pattern	**1.55 (1.35, 1.77)** ***
Encouraged them to talk to a healthcare professional	0.94 (0.88, 1.01) *
Avoided talking about it	**2.08 (1.53, 2.84)** ***
Replacement ratio (OECD)	**1.03 (1.01, 1.06)** ***

a In this case, we examine respondents who endorsed ‘It would make other employees feel uncomfortable in response to the survey question: If someone in your workplace suffered with depression, what impact, if any, do you think it would have?

* = p<0.05,

** = p<0.01,

*** = p<0.001.

### Taking time off work as a result of depression

Table 4 describes the individual characteristics and country contextual characteristics associated with greater likelihood of employees with depression taking time off. In terms of individual characteristics, individuals with a university education were less likely than individuals without university education to report taking time off from work when they had a diagnosis of depression. In terms of country contextual characteristics, on average living in a country with a greater prevalence of managers saying that they encouraged the employee to talk to a healthcare professional was associated with a greater likelihood of taking time off from work. On average, living in a country with a greater prevalence of managers saying that they offered help, offered a flexible working pattern or avoided talking to the employee with depression was associated with a lower likelihood of taking time off from work. On average, living in a country with a more generous benefits scheme was associated with a lower likelihood of taking time off from work.

**Table 4:** Individual, manager and country contextual characteristics associated with greater likelihood of employees taking time off as a result of depression (Multivariable logistic regression, n = 1,412).

	Adjusted GEE parameter estimates Odds Ratio (95% CI)
**Individual characteristics**	
Gender	
Female	0.84 (0.67, 1.06)
Male	Reference
Age	
45–64	1.25 (0.80, 1.93)
25–44	1.20 (0.81, 1.77)
16–24	Reference
University education	
Yes	**0.75 (0.61, 0.92)** **
No	Reference
Working in a larger company	1.05 (0.99, 1.11)
**Country contextual characteristics**	
Country prevalence of manager reactions to someone with depression	
Offered help to employee	**0.75 (0.70, 0.81)** ***
Offered flexible working pattern	**0.58 (0.53, 0.62)** ***
Encouraged them to talk to a healthcare professional	**1.21 (1.08, 1.35)** **
Avoided talking about it	**0.34 (0.28, 0.41)** ***
Replacement ratio (OECD)	**0.95 (0.94, 0.97)** ***

* = p<0.05,

** = p<0.01,

*** = p<0.001.

### Disclosure of depression in the workplace

Table 5 describes which individual characteristics and which country contextual characteristics are associated with greater likelihood of disclosing a depression diagnosis to an employer following time taken off from work. In regards to individual characteristics, females, older individuals (relative to 16–24 year olds) and individuals working in a larger company were more likely to tell their employer that they took time off work as a result of depression. Individuals with a university education were less likely than individuals without university education to tell employers. In regards to country contextual characteristics, on average living in a country with a greater prevalence of managers saying that they offered help to an employee was associated with a greater likelihood of disclosing depression to an employer, while a greater prevalence of managers saying that they encouraged the employee to talk to a healthcare professional or offered a flexible working pattern was associated with a lower likelihood of disclosure. On average, living in a country with a more generous benefits scheme was associated with a higher likelihood of disclosure.

**Table 5:** Individual, manager and country contextual characteristics associated with greater likelihood of disclosure to employer among employees with depression who took time off (Multivariable logistic regression, n = 1,412).

	Adjusted GEE parameter estimates Odds Ratio (95% CI)
**Individual characteristics**	
Gender	
Female	**1.49 (1.26, 1.75)** ***
Male	Reference
Age	
45–64	**3.06 (2.16, 4.31)** ***
25–44	**2.05 (1.57, 2.69)** ***
16–24	Reference
University education	
Yes	**0.50 (0.33, 0.76)** ***
No	Reference
Working in a larger company	**1.31 (1.22, 1.40)** ***
**Country contextual characteristics**	
Country prevalence of manager reactions to someone with depression	
Offered help to employee	**2.16 (1.97,2.36)** ***
Offered flexible working pattern	**0.50 (0.49, 0.51)** ***
Encouraged them to talk to a healthcare professional	**0.59 (0.57, 0.61)** ***
Avoided talking about it	1.03 (0.95, 1.12)
Replacement ratio (OECD)	**1.02 (1.01, 1.03)** *

* = p<0.05,

** = p<0.01,

*** = p<0.001.

## Discussion

Depression is experienced by a large proportion of the workforce and associated with high costs to employers; however, there is little research on factors which may influence the experience of having and coping with depression in the workplace and how this may vary by cultural setting across Europe. This study highlights the importance of both individual and sociocultural factors which may be associated with how people with depression are perceived and treated in the workplace, and hence, factors which may impact on openness and disclosure among employees with depression.

Our findings suggest that structural factors such as benefit systems and flexible working hours are important for workplace perceptions and employee outcomes; however, it seems that manager responses which focus on offering help to the employee with depression have the strongest association with positive perceptions in the workplace and also, openness and disclosure of employees with depression. Other research has emphasised the importance of positive attitudes in relation to social acceptance of people with mental illness as a key driver of stigma and has shown a direct link between these attitudes and the experiences of people with mental illness. For example, one study found that greater prevalence of comfort in talking to people with mental health problems among the public was associated with lower self-stigma, perceived discrimination and higher empowerment among people with mental health problems living in that country [22]. Social acceptance of people with depression; however, has not improved over the past 20 years [23] and research from Germany suggests that the public's unwillingness to recommend an individual with depression for a job increased between 2000 and 2011 (compared to 1990–2000) [24]. These attitudes could be critical for employment of people with mental illness, especially during a period of economic recession which can present disproportionate hardship for people with mental health problems [25].

Our study also identified other manager responses which were associated with employee outcomes and general workplace perceptions. A higher prevalence of managers avoiding talking with the employee about the problem was associated with a lower likelihood of taking time off work. This may indicate a general ignorance around depression. For instance, the data suggested that respondents lacked understanding of the symptoms and experience of depression, as respondents tended to associate depression more with affective symptoms, such as low mood, rather than cognitive symptoms, such as difficulty concentrating, indecisiveness and forgetfulness. Avoidance, however, may also result from prejudice and negative beliefs, and avoidance has been shown to be especially harmful in relation to employment of people with serious mental illness [26]; however, additional research is needed to better understand the dynamics of ‘avoidance’ in the workplace.

Interestingly, offering flexible working hours was also associated with a lower likelihood of taking time off work, a lower likelihood of disclosure and a higher likelihood of discomfort around depression in the workplace. Although it may be helpful for the employee to have the opportunity to work flexibly as they are recovering from an episode of depression, this strategy might also suggest that the problem could be solved in the workplace or through organisational strategies, and does not necessarily promote social inclusion or reduce stigma against people with depression. Importantly, a higher prevalence of managers encouraging employees to talk to a healthcare professional was the only factor associated with a higher likelihood of employees taking time off work as a result of their depression. It may be that this strategy signifies a culture which supports dealing with depression outside of the workplace through the support of health professionals. It is interesting to note that although flexibility in working arrangements and offering help or increased benefits may be recommended to support employees, they are not necessarily universally positive and so it may also be important to consider wider-ranging and indirect effects [27], [28] when implementing new policies. Other research suggests that independent of health, contextual and organizational factors may influence absenteeism and presenteeism, and these are not always associated in what is considered to be the expected direction. For instance, analysis in one study of qualitative interviews of individuals who had experienced mental health problems in the workplace suggested that although autonomy at work can facilitate control over workflow and working arrangements, this could increase the likelihood that one might stay in work as individuals might have the option to shorten the working day or adapt their tasks or working conditions depending on how they were feeling [28]. It suggests that these factors are complex and that flexibility could also lead to increased secrecy as there might be greater opportunity for concealment. Our findings also suggest that a culture which supports flexibility can be associated with reduced probability of taking time off work as a result of depression and a lower likelihood of disclosure to an employer. It could be that the outcome of reduced probability of taking time off work could vary according to context. For instance, offering help to the employee or flexible working arrangements may make the person feel that they can work around the issue in a way that does not require formal absence from work. Alternatively, under different circumstances, the employee might feel pressured to stay in work, for example, because of fear of losing their job. Interestingly, a culture where it is more likely for employers to offer help to employees in response to their depression was associated with a reduced likelihood of taking time off work (similar to a flexible working culture); but, a higher likelihood of disclosure (in contrast to a flexible working culture). In this case, employees may feel supported to stay at work and/or adapt their working style, but also more comfortable about discussing their depression with a supportive employer. Future research would benefit from collecting data on, for example the severity of depression, levels of presenteeism and explanations for why employees did or did not take time off work or disclose their depression in order to further contextualize these findings.

In terms of individual characteristics, females were more likely to feel comfortable with the issue of depression in the workplace and also to disclose their own depression to their employer. This is in line with previous research which suggests that females tend to have less stigmatising attitudes about people with mental illness [29]. Interestingly, individuals with a university education tended to be less likely to take time off work because of their depression or to disclose their depression to their employer. Although other studies have suggested that university education may be a protective factor in relation to self-stigma [22], some research suggests that individuals categorised as having a higher socioeconomic position are less likely to disclose a (hypothetical) mental illness [30].

To understand the social impact of depression in the workplace, it is important to investigate perceptions of employees and managers alongside experiences of employees with depression and their relationship. For example, knowing someone with a mental illness is associated with better attitudes and less discriminatory behaviour; however, it is contingent upon disclosure of a mental illness, which is also influenced by social acceptance [31]. Other research has suggested a need for better understanding of how societal beliefs and employment context influence the experience of depression and potential for disclosure [32].

This study begins to fill an evidence gap by identifying important societal factors which promote positive perceptions about people with depression in addition to openness and disclosure. A recent review of the literature identified nine factors associated with disclosure of a mental illness in an employment setting; however, all of the identified studies were from the USA and they tended to focus on individual factors (e.g., gender, severity of symptoms, diagnosis) or were performed within supported employment rather than mainstream employment settings [33]. One organisational factor - working in a mental health setting rather than another type of work setting - was associated with disclosure [34]; but little information is available on how societal factors may influence both the experience of and responses to depression in the work place. Our research provides initial evidence that in addition to individual factors, there are important contextual factors related to employment setting including manager responses and support in addition to benefit structures which might be important for how depression is experienced in the workplace.

### Strengths/Limitations

This study addresses a gap in the literature in terms of developing our understanding of social and cultural factors associated with depression in the workplace. Our findings come from a unique dataset including both employees and managers from seven countries across Europe, and information on their personal experiences of depression or their general perceptions of depression in the workplace. Although diagnosis of depression was based on self-report and we were not able to control for clinical characteristics, such as severity and/or type of symptoms, the characteristics of respondents with or without depression are in line with other epidemiological research. For instance, study respondents reporting a diagnosis of depression were more likely to be female, divorced and working part time. Individuals who reported never having a diagnosis of depression were more likely to be married, in the youngest age group (16–24) and working full time [9], [35], [36]. Survey responses also suggested differences in prevalence of depression and attitudes towards people with depression by country. In this study, reported prevalence of depression in the workplace varied by setting, with female and male respondents from Italy being significantly less likely to report having a diagnosis of depression compared to respondents from Great Britain or from Turkey. Data from the ESEMED (European Study of the Epidemiology of Mental Disorders) study also demonstrated lower prevalence rates of all major diagnostic groupings, including mood disorders, in Italy compared to five other countries in Europe (Belgium, France, Spain, The Netherlands and Germany), though the differences in magnitude were smaller in the ESEMED study [37].

Additional limitations are that data from this study did not include information on variables such as ethnicity or migration which might also be related to social exclusion in employment settings, in addition to mental illness and a low response rate. This study lacks detail on clinical characteristics, functioning and work roles, meaning that we could not explore how these might be related to consequences of or reactions to depression in the workplace. For instance, it could be that the consequences of certain workplace attitudes and/or practices might differ by severity of depression and future research might explore the complexity of these relationships and whether, for example, openness and support might be more important for someone who experiences chronic episodes of depression. As we include a mixture of aggregate country characteristics in addition to individual characteristics, this is a partial ecological study. Although we feel that it is important to explore the relationship between individual and cultural factors in this case, the results should be interpreted with due caution.

A strength of the study is that it draws on data from seven countries, but this does not necessarily mean that the findings are generalisable. Finally, these data were cross-sectional, so it was not possible to examine the pathway or mechanism by which, for example, disclosure or manager response is related to workplace perceptions or directly impacts on an employee with depression.

### Conclusion

Previous research has noted that absenteeism and early retirement as a result of mental illness, especially depression, seem to be increasing across Europe [38]. Our research highlights the potential role of cultural and organisational characteristics, especially around support and social acceptance of employees; these factors may influence the experience of and consequences for employees with depression, and hence, could also be important for productivity. Some responses, such as flexible working hours, may be helpful but are not necessarily sufficient, and our findings also emphasise the importance of support and openness of managers in addition to flexible working hours. Improving workplace attitudes and providing a supportive environment in which an employee can feel comfortable to disclose their depression may be one pathway toward improving social acceptance of employees with depression. Given the associated increases in absenteeism, this should be an important consideration for employers. This is especially important in light of recent evidence which suggests that social acceptance of people with depression is not improving [23] and thus it is likely that targeted efforts are needed [39] to address social acceptance of people with depression in the workplace.
